# Filaggrin^High^ melanomas exhibit active FGFR and allergic signatures with impaired GNA14 and Th1 signatures

**DOI:** 10.3389/fgene.2025.1569403

**Published:** 2025-07-18

**Authors:** Goodwin G. Jinesh, Isha Godwin

**Affiliations:** ^1^ Department of Molecular Oncology, H. Lee Moffitt Cancer Center & Research Institute, Tampa, FL, United States; ^2^ Molecular Medicine Program, H. Lee Moffitt Cancer Center & Research Institute, Tampa, FL, United States; ^3^ Saveetha Medical College, Chennai, Tamil Nadu, India

**Keywords:** filaggrin, pruritus, GNA14, IFN-γ, Th1 signaling, *Staphylococcus*, human papilloma virus

## Abstract

Filaggrin gene (*FLG/FLG2*) product deregulations are associated with various allergic skin diseases, including but not limited to atopic dermatitis, alopecia areata, and ichthyosis vulgaris. However, the molecular immunological underpinnings of filaggrin phenotype manifestations are not completely understood. To gain insight into the underlying context, we classified the melanomas based on the filaggrin expression (filaggrin^High/Low^) and profiled the signaling context behind pruritic melanomas. We identified that the major signaling context changes behind filaggrin^High^ melanomas are active FGFR signaling and impaired GNA14 and Th1 signatures, in addition to many genetic and immune changes that are associated with pruritus.

## Introduction

Filaggrin gene (*FLG/FLG2*) mutations and expression are associated with various allergic skin diseases, such as atopic dermatitis, alopecia areata, and ichthyosis vulgaris (Smith et al., 2006; Irvine et al., 2011). These diseases are characterized by the terminal differentiation of keratinocytes and skin cells into keratinized and cornified envelope-enriched layers of skin as a result of pruritic allergies. However, the molecular signaling underpinnings of these skin phenotype manifestations behind filaggrin are poorly understood. Filaggrins also affect the biology of melanoma angiogenesis and malignancy, which primarily target the skin (Thyssen et al., 2018; Leick et al., 2019). Pruritus in melanoma patients is induced in the context of checkpoint immunotherapy (Salinas et al., 2021). Pruritus in the skin is an itching sensation, and the scratch response is triggered by microbial and/or autoantigens through an immune cell-directed allergic reaction involving histamine (Yarwood et al., 2000). Histamine has the potential to inhibit interferon-γ (IFN-γ) signaling in melanoma (Kanda and Watanabe, 2002) and, therefore, could blunt Th1 immunity (Bradley et al., 1996). Interestingly, individuals with the loss-of-function variations in *FLG* are diagnosed with melanoma frequently (Adelanwa et al., 2022). To gain insight into the signaling context behind filaggrins, we examined the filaggrin expression (filaggrin^High/Low^: combined FLG and FLG2 mRNAs; see the methods section for details)-associated melanoma transcriptome to understand the underlying genetic and immune-signature context behind pruritic melanomas.

## Methods

### TCGA skin cutaneous melanoma (SKCM) data patient grouping based on filaggrin

Skin cutaneous melanoma (SKCM) RNA-Seq data were obtained from the Cancer Genome Atlas (TCGA) (https://gdac.broadinstitute.org/). The RNA-Seq dataset was processed as described previously (Jinesh et al., 2024a). Briefly, the RNA-Seq dataset was sorted based on FLG, FLG2, or a combination of FLG + FLG2 mRNA expression to stratify patients into FLG^High^ versus FLG^Low^ (n = 73 each), FLG2^High^ versus FLG2^Low^ (n = 73 each), and FLG + FLG2 filaggrin^High^ versus FLG + FLG2 filaggrin^Low^ (n = 47 each) groups of equal size within sets to have equal statistical power. Further details regarding the number of patients per group are given in specific sections below.

### Patient overall survival analysis of filaggrin-based melanoma patient groups

The overall survival data of the filaggrin-based patient groups were analyzed as described previously (Jinesh et al., 2021; Jinesh et al., 2020; Jinesh and Kamat, 2017). Briefly, TCGA SKCM survival data for FLG mRNA expression-based groups were obtained through OncoLnc (http://www.oncolnc.org/) using 16% settings for the FLG^High^ and FLG^Low^ groups (n = 73 each) to get the survival data. Similarly, FLG2 mRNA expression-based groups were obtained using 16% settings for the FLG2^High^ and FLG2^Low^ groups (n = 73 each) to get the survival data. The FLG + FLG2 mRNA expression-based groups were obtained after calculating the average of FLG and FLG2 mRNA expression from RNA-Seq data and sorting based on the average mRNA expression of FLG and FLG2 mRNAs per patient. Then, the group was set at >90 versus <10 percentiles for the FLG + FLG2 filaggrin^High^ and FLG + FLG2 filaggrin^Low^ groups (n = 47 each) to get the survival data (Supplementary Figure S1A) (see the statistics section for significance calculations).

### SKCM FLG versus FLG2 mRNA expression correlation analysis

SKCM RNA-Seq data from TCGA SKCM samples were processed to classify the normal versus tumor, and the tumor sample set was used to create an XY-plot of the mRNA expression data of FLG and FLG2 using GraphPad Prism v.7.04 (La Jolla, CA, United States). The Pearson *r* ^2^ correlation coefficient was calculated at a 95% confidence interval using the GraphPad software.

### cBioPortal: SKCM FLG and FLG2 mutation analysis

SKCM mutation data for FLG and FLG2 were obtained through cBioPortal (https://www.cbioportal.org/) (Cerami et al., 2012; Gao et al., 2013). Briefly, SKCM FLG + FLG2 filaggrin^High^ and filaggrin^Low^ group patient IDs were fed into cBioPortal as groups, and the mutation data for driver mutations and variants of unknown significance (VUS) were examined. The data were presented in a tabular form. There was no significant difference in mutation numbers.

### cBioPortal: SKCM 450 k methylation data analysis

Methylation analysis was carried out as described previously (Jinesh et al., 2018), with minor modifications. Briefly, the 450 k methylation data of TCGA SKCM samples were accessed through cBioPortal (https://www.cbioportal.org/) (Cerami et al., 2012; Gao et al., 2013). The methylation log_2_ data and their negative log_10_ p-value data were then used to create an XY-plot in GraphPad Prism v.7.04 (La Jolla, CA, United States). Datapoints with moderate (p=<0.05 to >0.001) to high (p=<0.001) significance on the high- and low- methylated genes were color-coded, as indicated in the figure.

### Gene set enrichment analysis (GSEA) and genomic positional GSEA

GSEA was performed as described previously (Jinesh et al., 2024b). Briefly, the SKCM RNA-Seq data from the filaggrin^High^ and filaggrin^Low^ groups were subjected to FPKM adjustment, and group averages for all genes were calculated. The filaggrin^High^ and filaggrin^Low^ group averages were subjected to GSEA for Hallmark, C1-positional, C2-curated, C3-motif, C5-Gene Ontology, C6-oncogenic, C7-immunologic, and C8-cell type complete gene-set collections/modules (MsigDB: https://www.gsea-msigdb.org/gsea/msigdb/ against Human_Illumina_Array_MSigDB.v7.5.1.chip). The phenotypes analyzed were filaggrin^High^ and filaggrin^Low^ tumors (switching class-B: filaggrin^High^ versus class-A filaggrin^Low^ phenotype), the >21,990 normalized enrichment scores (NESs) of all modules that were run (excluding C1-positional, which was processed separately) were combined in Microsoft Windows 10 PowerShell, and the top ranks were identified based on NES sorting.

The top-ranked gene sets with the lowest FDRq value and NES >2 or < −2 were used to generate a dotplot using the ggplot2 package in R. The FDRq values were converted to negative integers to reflect false discoveries as small dots in the dotplot. The dotplot and scale were composited in Adobe Photoshop CS5 and resized simultaneously using the lock aspect ratio to avoid unintended changes in dot sizes of the plot and the scale.

The C1-positional set enrichments were sorted based on NESs, and the top upregulated signature was examined for filaggrins and associated gene identities using GraphPad Prism v.7.04 (La Jolla, CA, United States).

R code:

library(ggplot2)
ggplot(DFname, aes(x = Xgroup, y = YGeneset)) +
geom_point(aes(size = NegativeFRDqValue, color = NES)) +
scale_color_gradientn(colors = c(“magenta,” “steelblue,” “black,” “white,” “brown,” “red,” “yellow”),
limits = c(-3, 3))

### Network analysis of differentially expressed genes between the filaggrin^High^ and filaggrin^Low^ groups

The differentially expressed genes between the filaggrin^High^ and filaggrin^Low^ groups were filtered based on the p-value (cut off: <0.05) and log_2_ fold change (>2 for genes upregulated in filaggrin^High^ melanomas and <2 for genes downregulated in filaggrin^High^ melanomas). The gene symbols of the 536 upregulated genes in filaggrin^High^ melanomas were fed into the NetworkAnalyst web server (https://www.networkanalyst.ca/) (Zhou et al., 2019) using the gene list input mode, and the analysis was run using the SIGNOR 2.0 database of Signaling Network. The network was organized into a circular/bi/tripartite layout before exporting the image. A similar analysis was carried out for 116 downregulated genes in filaggrin^High^ melanomas. Node tables were exported for both the upregulated and downregulated gene sets, and the degree and betweenness scores were fed into the ggplot2 package in R to generate ranked dotplots to see the top-node-associated genes.

R codes:

> library(ggplot2)
# For upregulated genes in filaggrin^High^ melanomas
> ggplot(DFname, aes(x = XGroup, y = YGene)) +
geom_point(aes(size = Betweenness, color = Degree)) +
scale_color_gradientn(colors = c(“black,” “blue,” “magenta,” “red”),
limits = c(2, 32))
# For downregulated genes in filaggrin^High^ melanomas
> ggplot(DFname, aes(x = XGroup, y = YGene)) +
geom_point(aes(size = Betweenness, color = Degree)) +
scale_color_gradientn(colors = c(“black,” “blue,” “magenta,” “red”), limits = c(0, 100))

The dotplot and scale were composited in Adobe Photoshop CS5 and resized simultaneously using the lock aspect ratio to keep the dot size matching between the plot and the scale.

### Detailed translation of the filaggrin-based transcriptome in Enrichr

Differentially expressed genes between the filaggrin^High^ and filaggrin^Low^ groups that were filtered based on the p-value and log2 fold change and sorted as upregulated and downregulated genes for NetworkAnalyst analysis (see above in the network analysis section) were fed into the Enrichr online web server (Chen et al., 2013; Kuleshov et al., 2016; Xie et al., 2021) to examine the top signatures based on the expression of these genes. All Enrichr signatures were collected as text files, merged in Windows 10 PowerShell, and sorted based on adjusted p-values. The top signatures obtained for the upregulated and downregulated signature results were categorized into disease, tissue, gene co-expression, genetics, microbes and immunity, and chemicals and drugs. The adjusted p-values and the number of gene hits for the categorized signatures were plotted as dotplots using the ggplot2 package in R. The dotplot and scale were composited in Adobe Photoshop CS5 and resized simultaneously using the lock aspect ratio to avoid unintended changes in dot sizes of the plot and the scale. To make the dot sizes comparable between dot plots of various categories, minimum and maximum limits were set based on the full dataset range. The minimum and maximum limits of the dots were cropped off during figure compositing.

R code:

> library(ggplot2)
# For upregulated genes in filaggrin^High^ melanomas
> ggplot(DFname, aes(x = Xgroup, y = YGeneset)) +
geom_point(aes (size = GeneNumber, color = AdjpValueR)) +
scale_color_gradientn (colors = c(“black,” “red,” “yellow”), limits = c(0, 300))
#GeneNumber = Number of hits
# For downregulated genes in filaggrin^High^ melanomas
> ggplot(DFname, aes(x = Xgroup, y = YGeneset)) +
geom_point(aes(size = GeneNumber, color = AdjpValueR)) +
scale_color_gradientn(colors = c(“black,” “magenta,” “skyblue1”), limits = c(0, 30))
#GeneNumber = Number of hits

### Statistical analyses

The filaggrin-based group survival data were then plotted using GraphPad Prism v.7.04 (La Jolla, CA, United States), and the log-rank (Mantel–Cox test) p-values were considered for the level of significance, as described previously (Jinesh et al., 2017). P-values <0.05 were considered minimally significant, and those <0.001 were considered highly significant. The sample number “n” for the TCGA data patient groups is indicated in the figures. For the methylation analysis, the p-value of 0.05 was considered significant, and p-values <0.001 were considered to be of robust significance. The p-values were based on cBioPortal analysis. For Enrichr dotplots, adjusted p-values were considered for significance inferences. For GSEA, normalized enrichment scores (NES) with the lowest FDRq values (close to zero) were considered as strong enrichments (negative or positive).

## Results

**FIGURE 1:**
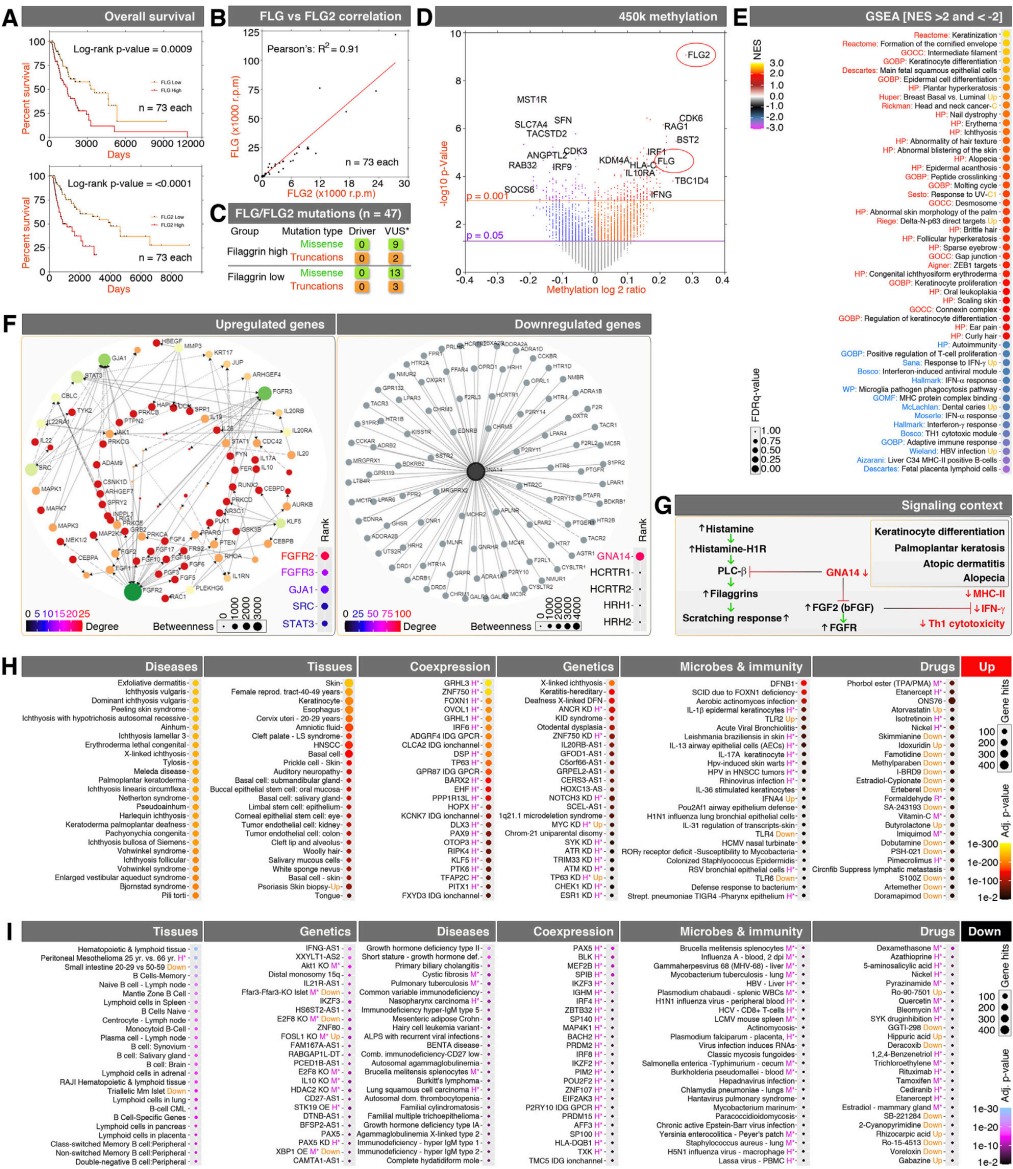
Filaggrin-based expression profiling reveals the underpinnings of the signaling and immune context of filaggrin^High^ melanomas. **(A)** Kaplan–Meier curves of FLG (top) and FLG2 (bottom)-based classifications of melanoma. **(B)** Correlation analysis of FLG and FLG2 expression in melanomas. **(C)** cBioPortal-based driver and variants of unknown significance (*VUS) mutations between filaggrin^High/Low^ groups of melanomas. **(D)** Methylation versus mRNA expression volcano plot showing FLG and FLG2 (red ellipse marks) at the highly methylated subset. **(E)** GSEA of filaggrin^High/Low^ RNA-Seq data. **(F)** Network analysis of filaggrin^High/Low^ RNA-Seq data to get the signaling connections. The color scale and dot size are for the dotplot on the bottom right, showing the top-ranked signaling networks. **(G)** Schematic showing the signaling context inferred using panel-**f** and the published literature. Red indicates inhibited or downregulated signaling components. Green or black indicates upregulated signaling components/phenotypes. **(H–I)** Detailed Enrichr profiling of the filaggrin^High/Low^ RNA-Seq data for the upregulated genes panel **(H)** and downregulated genes panel **(I)**. Purple *H indicates human data, and *M indicates mouse data.

High filaggrin mRNA expression (*FLG* and/or *FLG2* transcripts) is associated with a poor overall survival in melanoma (Figure 1A), and a positive correlation was observed in the expression patterns between the *FLG* and *FLG2* transcripts (Figure 1B). Therefore, based on the average of the combined FLG and FLG2 mRNA expressions, we classified the melanoma patients into the filaggrin^High^ and filaggrin^Low^ groups, respectively (Supplementary Figure S1A). Interestingly, no recognizable mutation status differences were detected between the groups (Figure 1C), but significant methylation log2 ratio differences for the *FLG* and *FLG2* genes were found (Figure 1D).

GSEA examination of differentially expressed genes between the filaggrin^High^ and filaggrin^Low^ groups confirmed the enrichment of RNAs related to clinically observed known human phenotypes driven by filaggrins, such as atopic dermatitis, alopecia, and ichthyosis, in addition to cell–cell contact structures, skin keratinization, and keratinocyte differentiation signatures (Figure 1E). Consistently, filaggrins co-expressed with the cornified envelope-regulatory gene signature (Supplementary Figure S1B). Conversely, a notable suppression of the interferon, major histocompatibility complex II (MHC-II), and Th1 cytotoxic modules was evident at the RNA level (Figure 1E). These data suggest that the suppression of the Th1 cytotoxic immunity signature is associated with the ichthyosis-related gene expression signature in filaggrin^High^ melanomas.

Network analysis of the top significantly upregulated genes displayed a large network of the FGF–FGFR system, and the significantly downregulated genes displayed a large network of GNA14-networked genes (Figure 1F). Based on the known interactions between the FGF–FGFR system versus the GNA14 system, as described by a previous study (Zou et al., 2019), we inferred the underlying signaling context that a loss of the GNA14–GPCR system might activate the FGF–FGFR system in filaggrin^High^ melanomas, and FGF signaling could further counteract interferon signaling (Jinesh et al., 2020; Maddaluno et al., 2020) to disable the Th1 cytotoxic module (Bradley et al., 1996) (Figure–1G).

As the known phenotypes were consistent with the gene network context, we carried out a detailed translation profiling of the filaggrin^High^ melanomas using upregulated (Figure 1H) and downregulated (Figure 1I) genes in Enrichr and categorized the phenotypes into disease, tissues, co-expressed genes/products, genetic factors, microbes and immunity, and chemical drugs that potentially regulate these gene expression patterns to accentuate the potential causes of filaggrin^High^ phenotypes.

In the upregulated gene signatures in filaggrin^High^ melanomas, the disease set revealed multiple notable skin exfoliating ichthyosis conditions and palmoplantar keratoderma (Figure 1H). The tissues further indicated that not just the skin but also woolly hair, esophagus, auditory nerves, and epithelial cells lining the alimentary canal and reproductive tract-related signatures were affected in filaggrin^High^ melanomas (Figure 1H). The genetic set implicates the X-chromosome involvement in the filaggrin^High^ melanomas while corroborating with keratinization and deafness (keratitis–ichthyosis–deafness syndrome: KID syndrome) (Figure 1H). Microbes and immunity set indicates a vital clue that an aerobic *actinomyces* infection and associated defensin-β1 could be the dominant etiological cause, which could be complemented by human papillomavirus [HPV; and head and neck squamous cell carcinoma (HNSCC), which further suggests HPV, and *staphylococcal* signatures (Figure–1H). No single drug was found to influence the majority of the upregulated genes, as the genes influenced by individual drugs were approximately just one-fourth of the total upregulated genes, but could knock out crucial drivers (such as transcription factors) to have a broader impact (Figure 1H).

On the downregulated gene set side in filaggrin^High^ melanomas, less pronounced gene enrichment was observed because fewer numbers of gene mRNAs were suppressed. However, these candidates were closely connected to lymphoid cell-mediated immunity, interferon-antisense, and so on (Figure 1I). The majority of other signature enrichments were only found to be influenced by a small set of genes despite significant scores, thus raising reliability issues; therefore, we listed those with a cautionary note (Figure 1I).

All these data, taken together show that a major change in FGF/FGFR versus GNA14/Th1 cytotoxic module gene expression underlies filaggrin^High^ melanomas, which blends with allergic skin disease signatures, such as atopic dermatitis, alopecia, and ichthyosis, in addition to cell–cell contact, skin keratinization, and keratinocyte differentiation.

## Discussion

Initial observations on the survival of melanoma patients stratified by filaggrin mRNA expression showed a significantly poorer survival in the filaggrin^High^ group. However, overall survival of melanoma patients can be influenced by numerous factors other than melanoma itself due to the unusual time it often takes for the disease to progress. However, one possible cause could be that an impaired Th1 response could expose patients to more infections and reduce their survival.

Interestingly, filaggrins displayed significant methylation along with interferon signaling component targets and still displayed contrasting expression patterns at the mRNA level, where the interferon signaling targets were downregulated but the filaggrins were upregulated. This difference suggests that the levels of filaggrin mRNAs might be regulated either at the post-transcriptional level to have increased stability or at the transcriptional level by exposing the genomic loci in euchromatin regions compared to the interferon-related loci. Furthermore, the lack of distinctive mutations between the groups rules out the possibility of mutation-based nonsense-mediated decay. Further studies are required to understand this notion.

Using GSEA, we first ascertained that the known filaggrin-related gene signatures, such as those associated with atopic dermatitis, alopecia, and ichthyosis, are also evident in filaggrin^High^ melanomas before carrying out detailed profiling and context inference. Based on GSEA, network analysis, and Enrichr profiling, we identified for the first time that upregulated FGF/FGFR, allergic signatures versus impaired GNA14, and Th1 signatures as the major changes in the context of filaggrin^High^ melanomas. This is possibly because of the microbiome in the melanoma that includes *Staphylococcus* (Mekadim et al., 2022), which is known to suppress IFN-γ signaling and Th1 cell recruitment (Li et al., 2017) to evade the immune system (Frodermann et al., 2011). The FGF/FGFR system, on the other hand, cooperates with this context as it is also known to inhibit IFN-γ signaling (Adachi et al., 2022), and inhibiting the FGF/FGFR system is required to boost checkpoint inhibitor cancer immunotherapy (Ruan et al., 2023).

The immune checkpoint therapy in melanomas induces pruritus in melanomas (Salinas et al., 2021; Fischer et al., 2013), which explains in part why filaggrin^High^ melanomas exhibit allergic signatures and suggest Th1 alterations. This allergic signature that is exhibited could be due to the microbiome involved in the melanomas. For example, melanomas are known to harbor discriminately abundant microbes, including *Staphylococcus* (Mekadim et al., 2022), which can produce IgE-inducing toxins (Bachert et al., 2020) and a protease-based allergic immune response (Krysko et al., 2019). Interestingly, this is similar to the allergic skin disease atopic dermatitis, where the lesions harbor discriminately abundant microbes, including *Staphylococcus* (Clausen et al., 2018). In support of our findings, histamine is known to inhibit interferon signaling in melanomas (Kanda and Watanabe, 2002), and checkpoint inhibition is known to induce a pruritic response in melanomas (Salinas et al., 2021; Fischer et al., 2013). Upregulation of defensins (DFNB1) further supports the involvement of microbes in pruritic melanomas.

Thus, for the first time, we identified a change in FGF/FGFR versus GNA14/Th1 signaling as the major underlying signaling context of filaggrin^High^ melanomas, which can be further explored through clinical and experimental studies. Our detailed profiling of filaggrin^High^ melanomas identifies additional potential causes of pruritic melanomas in the genetic (X-chromosome), microbial (bacteria and HPV), and immunological (Defensins) contexts.

## Data Availability

Publicly available datasets were analyzed in this study. These data can be found at TCGA: https://portal.gdc.cancer.gov/.
