# Supplementary material for: FilaggrinHigh melanomas exhibit active FGFR and allergic signatures with impaired GNA14 and Th1 signatures
Source: Front Genet. 2025 Jul 18;16:1569403. doi: 10.3389/fgene.2025.1569403 (PMC12322895; doi:10.3389/fgene.2025.1569403)
Supplement: Supplementary file 1 [file DataSheet1.doc]

**Supplementary information:** supplementary figure legends

**Filaggrin^High^ Melanomas Exhibit Active FGFR and Allergic Signatures with Impaired GNA14 and Th1 Signatures**

Goodwin G. Jinesh^1, 2,†,*^, and Isha Godwin^3,†^,

**Supplementary figure legend**

**Figure-S1 Filaggrin-based SKCM patient grouping, and C1-positional GSEA of differentially expressed genes. A,** Filaggrin-based SKCM patient grouping (FLG+FLG2 combined average-based) of melanoma (SKCM) tumor samples with equal number of samples in both groups (n = 47 samples each). A 0-10% and 90-100% ranges were set as cut-off for grouping. **B,** C1-positional GSEA of filaggrin^High^ versus filaggrin^Low^ differentially expressed genes showing the top enrichment of chromosome-1q21 band of human genome as the location with high differential gene expression. This gene signature is dominated by mRNAs of filaggrins, proline-rich proteins, late cornified envelope proteins, and S100A proteins.
