# Supplementary figures and images for: FilaggrinHigh melanomas exhibit active FGFR and allergic signatures with impaired GNA14 and Th1 signatures
Source: Front Genet. 2025 Jul 18;16:1569403. doi: 10.3389/fgene.2025.1569403 (PMC12322895; doi:10.3389/fgene.2025.1569403)

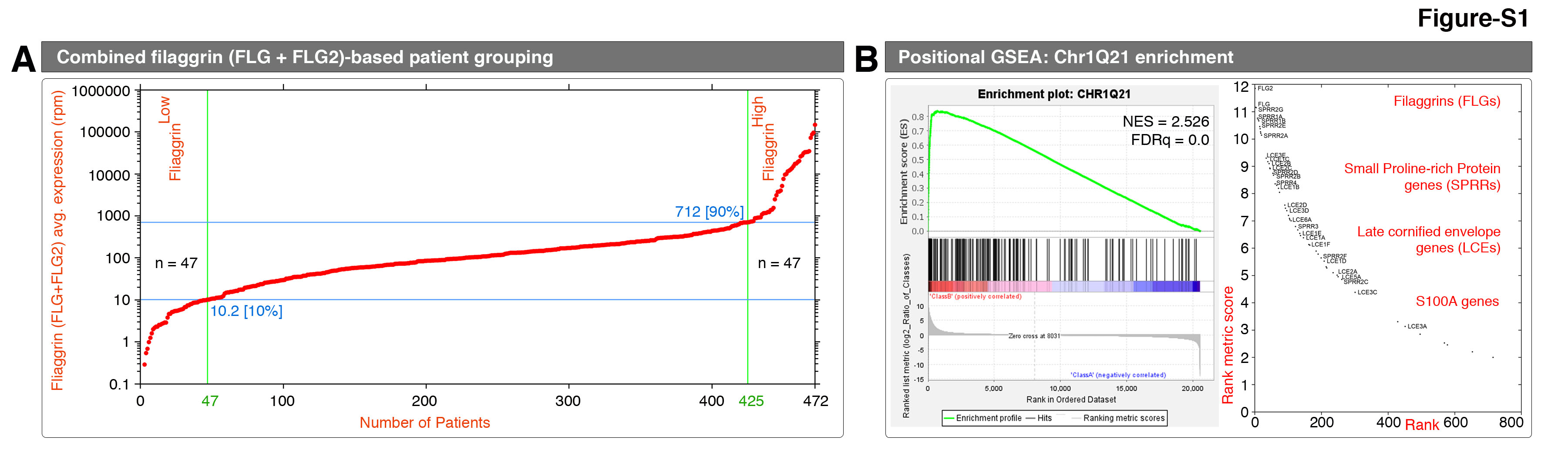

Supplement: Supplementary file 2 [file Image1.jpeg]
